# Arthrocentesis, Injectable Platelet-Rich Plasma and Combination of Both Protocols of Temporomandibular Joint Disorders Management: A Single-Blinded Randomized Clinical Trial

**DOI:** 10.7759/cureus.31396

**Published:** 2022-11-11

**Authors:** Wael Abbadi, Zafin Kara Beit, Nuraldeen M Al-Khanati

**Affiliations:** 1 Department of Oral and Maxillofacial Surgery, Faculty of Dental Medicine, Damascus University, Damascus, SYR; 2 Department of Oral and Maxillofacial Surgery, Faculty of Dentistry, Syrian Private University, Damascus, SYR

**Keywords:** joint crepitation, limited mouth opening, arthralgia, prolotherapy, platelet-rich plasma, arthrocentesis, degenerative disease, osteoarthritis, temporomandibular disorders, temporomandibular joint

## Abstract

Introduction

Osteoarthritis is one of the most common disorders of the temporomandibular joint (TMJ). The complex etiopathogenesis of the temporomandibular disorders (TMDs), and the variability of symptoms make it difficult to adopt standardized therapeutic protocols. Recently, platelet-rich plasma (PRP) injections have been applied into the TMJ in patients with TMJ osteoarthritis. On the other hand, arthrocentesis has received a widespread acceptance, as a minimally-invasive surgical procedure for TMDs. This study aimed to assess and compare the effect of each of these protocols (arthrocentesis, PRP injection, combination of them) in the management of TMJ osteoarthritis.

Methods

A single-blinded randomized clinical trial was conducted on a sample of 33 participants with limited mouth opening, pain on function and joint sounds due to TMJ osteoarthritis. Participants were assigned randomly into three groups: Arthrocentesis with PRP group; PRP group; Arthrocentesis group. Maximum mouth opening (MMO), pain and joint sounds were re-evaluated during a six-month observation period. Intra- and inter-group comparisons regarding these variables were performed.

Results

Participants in all study groups showed statistically significant improvement in terms of MMO and pain during the six-month postoperative period (p<0.05), and showed no significant improvements regarding joint sounds (p>0.05). Pain evaluations showed better outcomes in arthrocentesis with PRP group in comparison to arthrocentesis or injectable PRP alone after six months of follow-up (p<0.05). There were no significant differences in the outcomes of MMO and joint sounds evaluations between the three groups after six months.

Conclusions

Within the limitations of this study, it can be concluded that the three assessed treatment protocols were effective in improving limited mouth opening and pain in patients with TMJ osteoarthritis. A combination of TMJ arthrocentesis and PRP intra-articular injections showed the best outcomes regarding pain symptoms. None of the tested treatment protocols showed improvement in terms of articular sounds.

## Introduction

Temporomandibular joint (TMJ) is one of the most complex joints in the human body from the anatomical and physiological perspectives. TMJ is the only bilateral joint, where the right and left joints are fused and function together during mandibular movements. Moreover, TMJs comprise compound ginglymoarthrodial articulation between the mandibular condyles, articular discs, and glenoid fossae of the temporal bones [1]. TMJ is unique as ginglymoarthrodial joint because it shows both hinge and sliding movements.

A wide term has been evolved covering vast TMJ-related conditions, temporomandibular disorders (TMDs) [2]. TMDs were found to have a considerable and direct impact on patients’ quality of life [3]. Among the most common TMDs, inflammatory disorders exist where various joint tissues become inflamed as a result of insult or degradation of articular cartilage, e.g., in osteoarthritis [4]. Temporomandibular joint osteoarthritis (TMJ-OA) is a common chronic joint disorder that can be caused by micro- or macro-trauma to the TMJ, or by other pathological processes [5]. TMJ-OA leads to an inflammatory progressive degeneration of the TMJ articular cartilage [5]. Patients with TMJ-OA usually have dysfunction and pain in the TMJ region with a significant reduction in quality of life [6].

The treatment for TMJ-OA mainly includes non-surgical and minimal-invasive options, e.g., physical therapies, dietary adjustments, occlusal splints, oral non-steroidal anti-inflammatory drugs (NSAIDs), muscle relaxants, intra-articular drug therapies, and arthrocentesis [7, 8]. Arthrocentesis is a minimally invasive, simple, and effective therapy for TMDs [9]. Arthrocentesis is usually performed by inserting cannulas into the upper joint space at two different puncture sites [10]. This mainly aims to eliminate pain and inflammatory elements along with the synovial fluid from the upper TMJ space, and to reduce the friction between articular surfaces [10].

Platelet-rich plasma (PRP) is a biological therapy that comprises an autologous concentrate of platelets acquired by blood centrifugation [11]. This concentrate has shown potential benefits due to the abundance of growth factors within it. A systematic review in 2018 showed slight evidence for the potential superiority of PRP intra-articular injections in patients with TMJ-OA [12]. Arthrocentesis is considered a safe procedure that swiftly switches the TMJ from the "dysfunctional" to the "functional" state in patients with TMJ-OA [13]. Additional injection of PRP at the end of the arthrocentesis may provide better results than arthrocentesis alone [13]. These minimal-invasive approaches can be selectively considered in cases with failure of more conservative non-invasive approaches [4]. However, there is still controversy regarding which minimal-invasive method is most effective in TMJ-OA. Hereby, this study was conducted to compare the outcomes of three treatment protocols, namely arthrocentesis, PRP injection, and a combination of both arthrocentesis and PRP in relieving symptoms of TMJ-OA. The null hypothesis was that no differences exist between these treatment protocols in terms of pain, limited mouth opening, and joint sound improvement after six months of follow-up.

## Materials and methods

Study design and setting

A total of 33 participants were enrolled in this single-blinded randomized clinical study. Patients who developed a unilateral TMJ-OA were selected from cases admitted to the Department of Oral and Maxillofacial Surgery (Faculty of Dental Medicine, Damascus University, Damascus, Syria) in the period between April 2020 and September 2021. The study protocol was approved by the Research Ethics Committee of Damascus University (registration no. 2019-2504).

Sample size

Priori power analysis was performed using G*Power software version 3.1.9 (University Düsseldorf, Germany) for estimating the sample size for the current study. The total sample size required to detect a meaningful effect with a desired power of 95% was 27 participants. After considering a potential drop-out rate of 15%, the sample size was determined to be 33 participants (11 participants for each group).

Participants recruitment

All participants provided their consent prior to any intervention regarding this trial. Inclusion criteria included TMJ-OA patients aged more than 18 years, class I molar and canine relationship, baseline mouth opening less than 40 mm, unilateral TMJ pain with ipsilateral joint sounds, limited lateral mandibular movement (<8mm) in at least one direction, and poor masticatory efficacy. Poor masticatory efficacy was defined as participant’s inability to eat all kinds of solid food. Reported pain on palpation, pain on function, limited mouth opening, joint crepitation during function, and soft end feeling were the main clinical findings used to diagnose TMJ-OA in the present study. Exclusion criteria were rheumatoid diseases, history of TMJ surgery, articular adhesion, TMJ ankylosis, tumors of the condyle, history of condylar fracture, pregnancy, growth disorders, and bilateral joint pain and/or sounds. Participants with ipsilateral impacted, partially erupted, or buccoverted maxillary third molars were also excluded. Extraction of maxillary third molar, if indicated, should be done at least one month before recruitment. Also, patients who reported improvement of symptoms after other non-surgical treatments (i.e., physical therapy, muscle relaxant oral medication along with soft diet for two weeks) were not included in this study. Every participant had a thorough clinical examination to confirm the diagnosis of TMJ-OA, which included lateral palpation of the TMJ area with index fingers just anterior to the tragus. A standard palpation pressure of about 500g was implemented during TMJ palpation. Palpation was done by a surgeon who had received prior training on a scale to expertise in applying the standard digital palpation pressure during TMJ examination.

Randomization and allocation concealment

Participants were randomly assigned into three groups. Differences between study groups were based on the interventional treatment protocol to be implemented. Participants were either assigned to arthrocentesis with PRP treatment group, PRP only group, or arthrocentesis only group. Randomization was performed with aid of computer-generated random sequence. The allocation sequence was concealed in opaque, sequentially-numbered, sealed envelopes with a total of 33 envelopes. This was done by a third-party person who was not involved in the research project. Dark paper inside each envelope was used to deprive its permeability to light. Participants’ details (name and age) were written on the sealed envelopes that were opened only when it was time to allocate to treatment protocol after all baseline assessments were completed.

Baseline and outcome measures

All study participants had unilateral TMJ pain, ipsilateral joint sounds, and limited mouth opening at baseline. Baseline measurements of pain and maximum mouth opening (MMO) were done preoperatively. Pain was assessed by asking study participants to indicate the pain level on a 10-cm visual analog scale (VAS), where score 0 indicated “no pain at all”, and score 10 indicated “the worst pain ever”. Baseline MMO was measured as the distance in millimeters between the incisal edges of upper and lower incisors during maximal opening. Vernier caliper was used to measure MMO. Outcome measures were joint sounds (present/absent), MMO, and pain. Re-assessment of these variables was done in two follow-up timepoints.

Interventions

**Figure 1:**
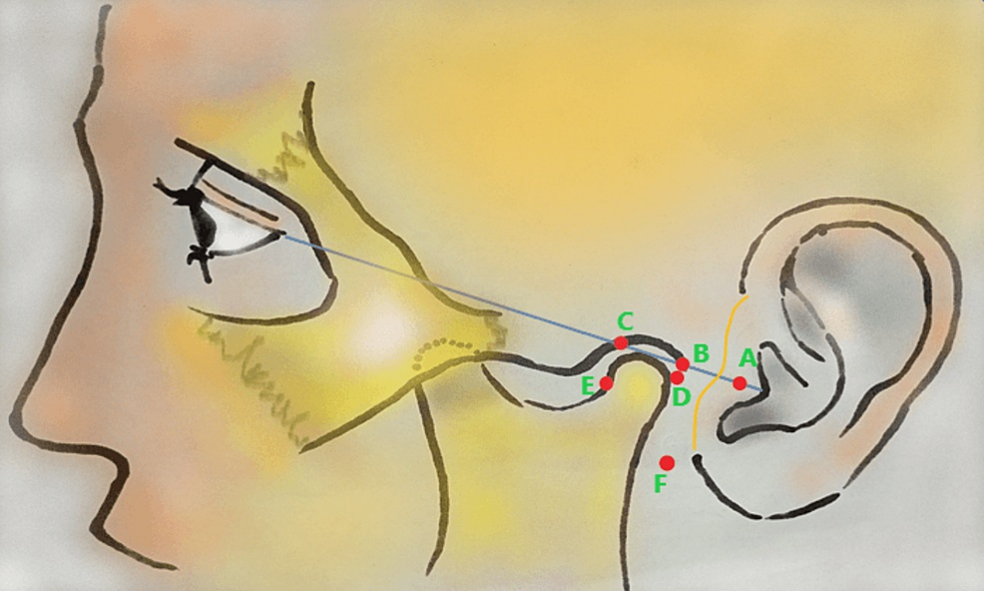
Surface landmarks and mark-points for intra-articular access in temporomandibular joint arthrocentesis and/or intra-articular platelet-rich plasma injection procedures. Canthal-tragus line (blue line); Preauricular line (orange line); Half distance from tragus tip to the intersecting point of blue and orange lines (point A); 10mm away from point A on the blue line (Point B); 10mm away from point B on the blue line (Point C); 2mm below point B (Point D); 10mm below point C (Point E); Auriculotemporal nerve block access point (Point F). This illustration is created by the authors of this study.

TMJ arthrocentesis procedures and related surface landmarks were in line with Lin et al. [13]. Procedures for all participants were done by the same team. Multiple facial surface landmarks were used to determine the anesthetic and cannula-puncture points (Figure 1). A line was drawn from the ipsilateral tragus to the lateral canthus. Also, a preauricular line was drawn just anterior to the pinna where pinna and facial skin met. Point A was in the mid-distance between the crossing point of the two aforementioned lines to the tragus tip. Point B was 10mm away from point A on the canthal-tragus line. Point C was 10mm away from point B on the same line. Point D was 2mm below point B on a line perpendicular to the canthal-tragus line. Point E was 10mm below point C likewise. Point F was 10mm anterior to the meeting point of ear lobule and facial skin.

Povidone-iodine 10% was used for skin disinfection preoperatively. Local anesthetic solution (lidocaine 2% with epinephrine 1:80,000) administration was done, and auriculotemporal nerve block was performed from Point F. A 27-gauge needle was inserted into point F and advanced until touching the posterior border of condylar neck. Negative aspiration before auriculotemporal nerve block was confirmed to avoid injecting into a blood vessel. For each participant in all study groups, 21-gauge cannulas were inserted into each of D and E points in superior, anterior, and inward directions to a depth of about 20mm while his/her mouth was open. Normal saline (1 ml) was injected through point D, while observing point E cannula to drain and ensure that the cannulas tips reached their target. Arthrocentesis of the TMJ was performed by injecting 50ml of saline solution over about 20min via point-D cannula allowing the elimination of synovial fluid and potential inflammatory mediators via the cannula at point E. In the PRP group, arthrocentesis was not performed. Instead, the point-E cannula was removed, and PRP was injected through the cannula at point D. Injectable PRP was prepared from venous blood (6ml), mixed with 3.2% sodium citrate as an anticoagulant, and centrifuged at 1000 rpm for 10 minutes. Intra-articular PRP injection (1ml) was performed in the PRP group and just after arthrocentesis in the “arthrocentesis with the PRP” group. In the arthrocentesis group, no intra-articular PRP prolotherapy was performed.

Follow-up

Patients were kept on a soft diet for one week. Paracetamol 500mg was prescribed thrice a day for five days. Patients' recall appointments were scheduled at one-month and six-month follow-up timepoints to re-evaluate pain, MMO, and joint sounds.

Statistical analysis

Data were collected, and SPSS version 19 (IBM Corp., Armonk, NY, USA) was used for statistical analysis. Regarding pain and MMO comparisons, one-way ANOVA was used for intergroup comparisons, and paired Student’s t-test for intragroup comparisons. For joint sounds, Chi-Square tests and McNemar tests were performed for intergroup and intragroup comparisons, respectively. Statistical significance was set at P<0.05.

## Results

**Figure 2:**
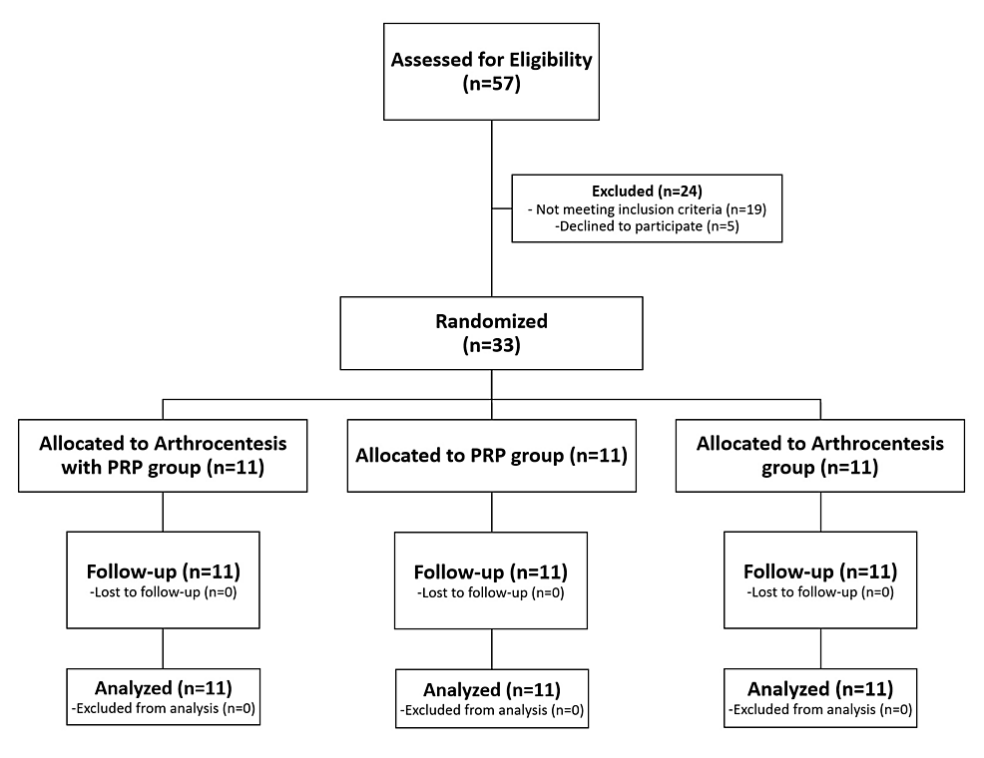
Flowchart presenting participants recruitment, allocation, follow-up, and analysis stages of this randomized clinical trial. A total of 57 patients (n=57) were assessed for eligibility: 24 patients (n=24) were excluded; five patients (n=5) declined to participate; 19 patients (n=19) did not meet the eligibility criteria (bilateral joint symptoms (n=7), symptoms improvement after non-invasive treatment protocol prior to enrollment (n=6), baseline maximum mouth opening of more than 40mm (n=4), and age less than 18 years (n=2)). A total of 33 participants (n=33) were included in all study phases.

Out of 57 potential participants who were initially evaluated, 33 participants (n=33) with unilateral TMJ-OA were allocated to study groups, and included in all phases of this trial (Figure 2). During eligibility assessment, five patients declined to participate, and 19 patients were excluded due to the following reasons: bilateral joint symptoms (n=7), symptoms improvement after other non-invasive treatments prior to enrollment (n=6), baseline MMO of more than 40mm (n=4), and age less than 18 years (n=2). 72.7% of participants were females (n=24) with sample mean age of 27.4 (±6.6) years. Osteoarthritis of right and left TMJs in the present study was nearly equal. Patients’ demographics and comparisons between the three study groups are presented in Table 1.

**Table 1:** Baseline demographics and characteristics of the study sample and intergroup comparisons. ^†^Analyzed by one-way ANOVA tests, ^‡^Analyzed by Chi-Square tests, A+PRP = Arthrocentesis with Platelet-Rich Plasma, PRP = Platelet-Rich Plasma, SD = Standard Deviation, % = Percentage, 1m = One-month follow-up timepoint, 6m = Six-month follow-up timepoint, VAS = Visual Analogue Scale, MMO = Maximum mouth opening (measured in millimeters).

		Protocols for temporomandibular joint osteoarthritis management					
		Total (n=33)		A+PRP Group (n=11)	PRP Group (n=11)	Arthrocentesis Group (n=11)	P-value
Age, years (mean±SD)		27.42 (±6.59)		26.45 (±4.66)	28.73 (±7.73)	27.09 (±7.38)	0.719^†^
Gender (n and %)							
	Male	9 (27.3%)		3	4	2	0.632^‡^
	Female	24 (72.7%)		8	7	9	
Surgical Side (n and %)							
	Right	17 (51.5%)		5	8	4	0.207^‡^
	Left	16 (48.5%)		6	3	7	
Joint Sounds- Baseline (n and %)							
	Present	33 (100%)		11	11	11	1.000^‡^
	Absent	0 (0%)		0	0	0	
Joint Sounds- 1m (n and %)							
	Present	21 (63.6%)		3	9	9	0.009^‡^
	Absent	12 (36.4%)		8	2	2	
Joint Sounds- 6m (n and %)							
	Present	31 (93.9%)		9	11	11	0.119^‡^
	Absent	2 (6.1%)		2	0	0	
VAS Pain Score- Baseline (mean ±SD)		4.73 (±1.99)		5.45 (±1.81)	4.18 (±1.72)	4.55 (±2.34)	0.312^†^
VAS Pain Score- 1m (mean ±SD)		1.45 (±1.00)		1.27 (±1.01)	1.18 (±0.60)	1.91 (±1.22)	0.182^†^
VAS Pain Score- 6m (mean ±SD)		1.91 (±1.40)		0.73 (±0.79)	2.18 (±1.25)	2.82 (±1.25)	0.000^†^
MMO- Baseline (mean ±SD)		36.15 (±3.19)		35.91 (±2.88)	35.82 (±2.86)	36.73 (±3.95)	0.774^†^
MMO- 1m (mean ±SD)		45.09 (±5.09)		48.82 (±4.19)	44.18 (±4.81)	42.27 (±4.15)	0.005^†^
MMO- 6m (mean ±SD)		46.18 (±6.56)		49.27 (±4.73)	46.09 (±7.05)	43.18 (±6.74)	0.090^†^

All study participants exhibited TMJ sounds, pain on function, and limited mouth opening (MMO less than 40m) at baseline. Intra-group comparisons showed significant differences in mean values between baseline preoperative measurements and after six months regarding VAS pain scores in all study groups (P<0.001, P=0.002, and P=0.008 for arthrocentesis with PRP group, PRP-only group, and arthrocentesis-only group, respectively). A similar trend was found regarding MMO in the three groups (P<0.001, P=0.001, and P=0.011 for MMO measurements within arthrocentesis with PRP group, PRP-only group, and arthrocentesis-only group respectively). No significant differences existed between preoperative and six-month postoperative assessments of joint sounds in all study groups (P>0.05). Intragroup differences analysis is shown in Table 2.

**Table 2:** Intragroup comparisons in term of pain, maximum mouth opening, joint sounds after six months of follow-up. ^†^Analyzed by Paired t tests, ^‡^Analyzed by McNemar Tests, A+PRP = Arthrocentesis with Platelet-Rich Plasma, PRP = Platelet-Rich Plasma, SD = Standard Deviation, VAS = Visual Analogue Scale, MMO = Maximum mouth opening (measured in millimeters).

	Group	Baseline	After 6 Months	P-Value
VAS Pain Score (mean±SD)	A+PRP Group	5.45 (±1.81)	0.73 (±0.79)	0.000^†^
	PRP Group	4.18 (±1.72)	2.18 (±1.25)	0.002^†^
	Arthrocentesis Group	4.55 (±2.34)	2.82 (±1.25)	0.008^†^
MMO (mean ±SD)	A+PRP Group	35.91 (±2.88)	49.27 (±4.73)	0.000^†^
	PRP Group	35.82 (±2.86)	46.09 (±7.05)	0.001^†^
	Arthrocentesis Group	36.73 (±3.95)	43.18 (±6.74)	0.011^†^
Joint Sounds- Present (n)	A+PRP Group	11	9	0.500^‡^
	PRP Group	11	11	1.000^‡^
	Arthrocentesis Group	11	11	1.000^‡^

Mean MMO value in arthrocentesis with PRP group one month postoperatively was 48.82 (±4.19) mm, compared to 44.18 (±4.81) mm and 42.27 (±4.15) mm in PRP-only and arthrocentesis-only groups respectively. This intergroup difference was statistically significant (P=0.005). However, there were no significant differences between the three groups in regard to MMO in the six-month follow-up timepoint (P=0.090). In contrast, mean VAS pain scores showed no significant differences between the study groups one month after the interventions (P=0.182). But, in the six-month follow-up timepoint, these differences in mean pain score between arthrocentesis with PRP group (0.73±0.79), PRP-only group (2.18±1.25), and arthrocentesis-only group (2.82±1.25) were significant (P<0.001). Intergroup differences are presented in Table 1.

Harms

All procedures were carried out without complications, except for one case where transient partial facial palsy occurred after anesthetic administration and infiltrated to a facial nerve branch.

## Discussion

Disorders related to TMJ have always been a thorny issue in the medical field. Symptoms of TMDs negatively affect patients’ quality of life to a great extent [14]. There is still controversy over the diagnostic methods and treatment protocols of different cases of TMDs. Promising results were seen by the implementation of combination therapy of more than one protocol in the management of TMDs [15]. In this study, three groups were compared in terms of clinical outcomes of different protocols of TMJ-OA management, namely TMJ arthrocentesis only, intra-articular PRP injection only, and arthrocentesis immediately followed by injectable PRP therapy. To the best of our knowledge, there was no previous study comparing all three aforementioned treatment protocols.

The following clinical variables were assessed at different timepoints: MMO, pain on function and joint sounds. It was found that MMO was improved in all study groups with no significant differences between the groups. Pain scores significantly decreased in all study groups in the short-term one-month follow-up with no intergroup differences. However, pain relief seemed to last longer in arthrocentesis with PRP treatment protocol group. The six-month follow-up showed significant intergroup differences in terms of pain indicating the advantage of the combination protocol over the other treatment protocols. This might be justified as a result of the synergy between TMJ arthrocentesis, which removed debris and inflammatory cytokines within the synovial fluid, reduced articular surface friction, and adjusted the hydraulic pressure within the articular cavity, with the PRP advantages that could aid in the healing process, analgesia, and elimination of inflammatory mediators, and could provide scaffold effect for stem cells migration [16-18]. Pain reduction after intra-articular PRP injection might be explained by the release of protease-activated receptors that possess potential anti-inflammatory and analgesic effect [19].

Only participants in the arthrocentesis-PRP group reported a significant reduction in terms of joint sounds one month postoperatively. Nevertheless, recurrence was observed sooner than expected with no significant joint sounds’ improvement at the six-month follow-up timepoint in comparison to the pretreatment status in all study groups. This might be explained by the potential presence of various influential psychological, social, economic and/or general health factors. This was in line with Moon et al. who found no significant improvement in joint sounds after PRP prolotherapy [20]. Derwich et al. conducted a systematic review in 2021, and concluded that TMJ arthrocentesis alone improved jaw function and significantly reduced pain symptoms in TMJ-OA cases [21]. They also reported that the results of additional injections of PRP at the end of the arthrocentesis were still questionable [21]. This provided justification for conducting the current study, which showed that these additional PRP injections could provide better pain and MMO outcomes.

However, this study had some limitations. The lack of a definitive assessment of the psychological state of study participants was one of the limitations. Although this was not applicable, taking into account the psychological state of the patients would make the outcomes more credible in representing the changes that occur during the trial. Also, a longer observation period for outcomes of treatment protocols would add a greater value in presenting and comparing the long-term effect of each TMJ-OA treatment protocol. Moreover, it was almost impossible to find TMJ-OA patients without parafunctional habits, e.g., bruxism. Therefore, bruxism and other parafunctional behaviors could not be excluded from the current study. Further, postoperative paracetamol and soft diet could have constituted as potential sources for bias. However, pain assessment was not performed in the first days when patients were on soft diet and allowed to take only paracetamol painkiller to control postoperative pain. Paracetamol was sufficient for pain control in all the patients, as the study interventional procedures were minimally invasive. Soft diet and painkillers were certainly not the causes of symptoms improvement because, as research methodology indicated, all participants were diagnosed with unilateral TMJ-OA that had failed to resolve by other non-invasive treatments including painkillers and dietary modifications prior to enrollment in the present study.

This study confirmed that TMJ arthrocentesis was a safe procedure with very limited complications. Out of the 22 cases that underwent arthrocentesis with or without PRP prolotherapy in this trial, only one case presented transient neurological complication related to the auriculotemporal nerve anesthesia. Complications that could develop during and after TMJ arthrocentesis were considered to be minimal and temporary [22]. These could include swelling of the periarticular tissues, ipsilateral temporary open bite, swelling of external auditory canal, and frontalis and/or orbicularis oculi paresis [22]. TMJ arthrocentesis procedure is minimal-invasive, nevertheless, great attention should be drawn to avoid vascular and neurological injuries, and to respect the upper TMJ thin lamina between the joint space and the neurocranial structures superiorly.

## Conclusions

Within the limitations of the current study, it can be concluded that it was beneficial to use arthrocentesis and/or PRP injection in the management of osteoarthritis symptoms. Arthrocentesis alone, PRP alone, and a combination protocol provided improved results in terms of the limited mouth opening and pain. Pain relief seemed to be better in the arthrocentesis-PRP treatment protocol. However, these protocols seemed to have no positive effect in the management of joint sounds in TMJ-OA.
